# IFN-γ Regulates the Expression of MICA in Human Corneal Epithelium Through miRNA4448 and NFκB

**DOI:** 10.3389/fimmu.2018.01530

**Published:** 2018-07-02

**Authors:** Dan Wu, Jing Zhang, Tingting Qian, Yiqin Dai, Alireza Mashaghi, Jianjiang Xu, Jiaxu Hong

**Affiliations:** ^1^Department of Ophthalmology, Eye, Ear, Nose, and Throat Hospital, Shanghai Medical College, Fudan University, Shanghai, China; ^2^Department of Immunology and Biotherapy Research Center, Shanghai Medical College, Fudan University, Shanghai, China; ^3^Leiden Academic Centre for Drug Research, Faculty of Mathematics and Natural Sciences, Leiden University, Leiden, Netherlands; ^4^Department of Ophthalmology, The Affiliated Hospital of Guizhou Medical University, Guiyang, China; ^5^Key Laboratory of Myopia, Ministry of Health (Fudan University), Shanghai, China; ^6^Shanghai Key Laboratory of Visual Impairment and Restoration, Fudan University, Shanghai, China

**Keywords:** IFN-γ, MICA, miRNA4448, NFκB, human corneal epithelium

## Abstract

**Purpose:**

Major histocompatibility complex class I-related chain A (MICA), a non-classical major histocompatibility complex molecule, can stimulate or co-stimulate CD8+ T cells or natural killer (nk) cells, thus affecting cornea allograft survival. This study investigated IFN-γ regulation of MICA expression levels in human corneal epithelium by miRNA4448.

**Methods:**

MICA expression levels in human corneal epithelial cells (HCECs) stimulated with IFN-γ were detected by qRT-PCR and an enzyme-linked immunosorbent assay, and differential miRNA expression levels were measured. qRT-PCR, Western blotting, and immunofluorescence staining revealed nuclear factor kappa B (NFκB)/P65 expression in IFN-γ-treated and miRNA4448-overexpressed HCECs. A luciferase reporter assay was used to predict the interaction between NFκB and MICA. Additionally, HCECs were transfected with MICA plasmid or treated with IFN-γ and NKG2D-mAb and cocultured with NK cells and CD8+ T cells. Cell apoptosis was measured using Annexin V/PI staining. qRT-PCR detected the expression of anti-apoptosis factor Survivin and apoptosis factor Caspase 3 in MICA-transfected and IFN-γ-treated HCECs after co-culturing with NK cells and CD8+ T cells.

**Results:**

IFN-γ (500 ng/ml, 24 h) upregulated MICA expression in HCECs *in vitro*. Among six differentially expressed microRNAs, miRNA4448 levels decreased the most after IFN-γ treatment. The overexpression of miRNA4448 decreased MICA expression. miRNA4448 downregulated NFκB/P65 expression in IFN-γ-induced HCEC, and it was determined that NFκB/P65 directly targeted MICA by binding to the promotor region. A coculture with NK cells and CD8+ T cells demonstrated that MICA overexpression enhanced HCEC apoptosis, which could be inhibited by NKG2D-mAb. Simultaneously, Survivin mRNA expression decreased and Caspase3 mRNA expression increased upon the interaction between MICA and NK (CD8+ T) cells in HCECs.

**Conclusion:**

IFN-γ enhances the expression of MICA in HCECs by modulating miRNA4448 and NFκB/P65 levels, thereby contributing to HCEC apoptosis induced by NK and CD8+ T cells. This discovery may lead to new insights into the pathogenesis of corneal allograft rejection.

## Introduction

Corneal transplantation is one of the most common human organ transplantations worldwide. Although the 1-year allograft survival rate is currently as high as 90%, more than half of transplantation patients suffer various types of corneal rejection, such as epithelial rejection, chronic stromal rejection, and endothelial rejection. To date, corneal rejection is the main cause of corneal transplant failure (1). Major histocompatibility complex (MHC) is thought to play a significant role in corneal immune status. However, MHC molecules are not expressed in normal corneal endothelial cells, and the restricted distribution of MHC molecules in corneal epithelial cells has called into question the role of MHC in corneal rejection (2). A large, multi-center follow-up study showed no significant correlation between the human leukocyte antigen, the gene complex encoding MHC, and clinical outcomes (3). Therefore, the role of other immune pathways (beyond the classical MHC antigen) in corneal allograft rejection requires further investigation.

Recently, natural killer (NK) and CD8+ T cells have been recognized as playing a role in corneal immune status. One study found that rejected corneal stroma and endothelium were infiltrated by NK cells, CD4+ T cells, and CD8+ T cells (4), and another found that rats with NK cell depletion experienced delayed corneal allograft rejection (5). CD8+ T cells have been demonstrated to mediate delayed-type hypersensitivity and cytotoxicity in corneal allografts (6). MHC class I-related chain A (MICA), a non-classical MHC molecule, can stimulate or co-stimulate CD8+ T cells or NK cells, thus affecting allograft survival (7). Many studies have demonstrated that IFN-γ affects MICA expression. Schwinn et al. (8) and Zhang et al. (9) confirmed that IFN-γ can decrease MICA gene expression in tumor cells, while Saikali et al. (10) found that MICA expression was upregulated after IFN-γ treatment for 2 days in microglia cells. In normal conditions, MICA is expressed in the cytoplasm of human corneal epithelial cells (HCECs) and plays a biological role by transferring to the cell surface or secreting into the extracellular matrix under inflammatory or stressful situations (11). We have previously shown that IFN-γ, an important trigger for corneal rejection, can upregulate MICA expression and enhance MICA-mediated cytolysis of human corneal epithelium via NK cells. However, the detailed mechanism remains unclear.

microRNAs (miRNAs) are defined as 18–25 nucleotide single-stranded RNA molecules endogenous in eukaryotes that can target messenger RNA (mRNA) by base pairing. This results in translational repression or mRNA degradation, thus regulating the expression of the target gene. Many efforts have been made to understand the function of miRNAs in the cornea. For example, Lee et al. found that miRNA-145 plays an important role in regulating corneal epithelial cell differentiation (12), and our previous study showed that miRNA-494 can inhibit NGF-induced cell proliferation by targeting cyclin D1 in HCECs. Interestingly, it was proven that IFN-γ can downregulate MICA expression *via* miRNA-520b, leading to the escape of tumor cells from immune surveillance (13). Therefore, we speculated that miRNAs might be critical mediators bridging MICA and IFN-γ in corneal immune status.

In this study, we performed a miRNA microarray to screen IFN-γ-related miRNAs in HCECs. The results show that miRNA4448 could influence MICA expression in IFN-γ-treated corneal epithelial cells by inhibiting the transcription factor nuclear factor kappa B (NFκB). The presence of MICA enhanced NK and CD8+ T cell-mediated cytotoxicity in corneal epithelium cells *via* MICA–NKG2D interaction, which damaged the target cells and threatened graft survival.

## Materials and Methods

### Cell Culture

The SV40-immortalized HCEC line was kindly provided by Dr. Kaoru Araki-Sasaki (14) (Osaka University, Osaka, Japan). HCECs were cultured in Dulbecco’s modified Eagle’s/F12 medium (DMEM/F12, HyClone; GE Healthcare Life Sciences, Logan, UT, USA) supplemented with 10% fetal bovine serum (FBS; Gibco; Thermo Fisher Scientific, Inc., Waltham, MA, USA) and 1% penicillin/streptomycin and were plated at a density of 1 × 10^5^ cells/cm^2^. The medium was changed every 2 days, and the cells were passaged until full differentiation was reached. Passage five cells were used in the experiment.

Peripheral blood mononuclear cells (PBMCs) were isolated from peripheral venous blood obtained from normal healthy volunteers, according to the instructions provided in the Human Lymphocyte Separation Medium manuscript (Biolegend, San Diego, CA, USA). Additionally, NK cells and CD8+ T cells were labeled by fluorescent antibody (NK cells: FITC-CD3^−^, APC-CD16^+^, RPE-CD56^+^; CD8+ T cells: FITC-CD8^+^) and were isolated from the PBMCs by flow cytometry sorting. They were then activated by IL-2 (Chiron, NC, USA; 50 U/ml for NK cells and 100 U/ml for CD8+ T cells).

In apoptosis experiments, NK cells and CD8+ T cells were cocultured with HCECs in DMEM/F12 complete medium. Before the coculture procedure, HCECs were seeded onto 24-well *U*-bottom plates for 12 h at 37°C. Effector (E) to target (T) ratios (E:T) were 6:1 for NK cells and 10:1 for CD8+ T cells. For blocking studies, NK and CD8 + T cells were pre-incubated for 30 min with 100 µg/ml of NKG2D-mAbs (Biolegend).

### Drug Treatment

To evaluate the concentration dependence, HCECs were treated with various doses of IFN-γ (100, 500, and 1,000 ng/ml) for 24 h, and the subsequent time-dependent experiments were performed using 500 ng/ml of IFN-γ, incubating for 1, 12, and 24 h. In NFκB-related experiments, HCECs were treated with 100 ng/ml pyrrolidine dithiocarbamate (PDTC) for 2, 4, 6, 8, and 10 h to determinate the time dependence. HCECs were then treated with different doses of PDTC (0, 10, 25, 50, 100, and 200 ng/ml) for 2 h.

### Cell Proliferation Assay

A Cell Counting Kit 8 (Dojindo, Kumamoto, Japan) was used to detect cell proliferation according to the user guidelines. Briefly, 1 × 10^3^ HCECs were added to 96-well-plates in 100 µl cell culture medium per well. The cells were then treated with 500 ng/ml IFN-γ for 24 h or not treated at all. Next, CCK8 solution was injected into each well for 1 h at 37°C until the color changed. The absorbance of each sample was determined at 450 nm using an Epoch microplate spectrophotometer (Bio Tek, Biotek Winooski, VT, USA) and a Synergy hybrid reader (Bio Tek). The assay was repeated in triplicate.

### Reverse Transcription Quantitative Polymerase Chain Reaction (RT-qPCR)

Total RNA was isolated from HCECs using Trizol (Invitrogen, Carlsbad, CA, USA) according to the manufacturer’s instructions. cDNA for miRNA and mRNA real-time PCR was synthesized using a One Step PrimeScript^®^ miRNA cDNA Synthesis kit (cat. no. D350A; Takara Biotechnology Co., Ltd., Dalian, China) and a PrimeScript™ RT reagent kit with gDNA Eraser (cat. no. RR047A; Takara Biotechnology Co., Ltd.), respectively. The SYBR^®^ Premix Ex Taq™ II Real-Time PCR kit (cat. no. DRR081A; Takara Biotechnology Co., Ltd.) was used with the ABI ViiA7 Real-Time PCR System (Thermo Fisher Scientific, Inc.) for the qPCR analysis. U6 RNA was used as a control for the miRNA, and GAPDH was used as a control for the mRNA. The primers used are listed in Table 1.

**Table 1:** RT-qPCR primer sequences.

Target gene/miRNA	Primer sequence
GAPDH	F: TGCACCACCAACTGCTTAGC
	R: GGCATGGACTGTGGTCATGAG
MICA	F: GAATCCGGCGTAGTCCTGAG
	R: TCCGGGGATAGAAGCTGGAA
NFKB/P65	F: ATGTGGAGATCATTGAGCAGC
	R: CCTGGTCCTGTGTAGCCATT
Survivin	F: TTCTGCTTCAAGGAGCTGG
	R: GCACTCTCCCAGTTTC
Caspase 3	F: GTGAGGCGGTTGTAGAAGAGTT
	R: TCACGGCCTGGGATTTCAAG
RNU6	F: CAAATTCGTGAAGCGTT
	R: TCA ACTGGTGTCGTG G
miRNA3136-5p	F: TGACTGAATAGGTAGGGTC
	R: CTCAACTGGTGTCGTG
miRNA4481	F: AGTGGGCTGGTGGTTC
	R: TCA ACTGGTGTCGTGG
miRNA4448	F: CTCCTTGGTCTAGGGGTA
	R: TCAACTGGTGTCGTGG
miRNA17-5p	F: TGTACAGCCTCCTAGCTT
	R: CAACTGGTGTCGTGGAG

### Enzyme-Linked Immunosorbent Assay (ELISA)

The amount of MICA present in the supernatant of the cell culture medium was detected using a human MICA ELISA kit (ProSpec-Tany; TechnoGene, Ness-Ziona, Israel) according to the manufacturer’s protocol.

### miRNA Microarray Analysis

IFN-γ was added to the culture medium at a final concentration of 500 ng/ml, incubating for 24 h. The total RNA amount was obtained according to the Trizol method and then sent to the MicroRNA Microarray Service (Gminix Biologic Science Co., Ltd, Shanghai, China), which used an AffymetrixGeneChip miRNA array. Differential miRNA expression was defined using a cut-off value of a twofold change and analyzed by Gene Ontology (GO; www.geneontology.org) using KEGG analysis and Ingenuity Pathway Analysis (IPA). mirBase, miRWalk, and targetScan bioinformatics software were used to predict the potential target genes of the miRNA.

### pLenti-CMV-GFP-miRNA4448 Transfection

The lentiviral vector, pLenti-CMV-miRNA4448, negative control (NC), pLenti-CMV-NC, and lentiviral packaging plasmids (Biofavor Biotechnology, Wuhan, China) were co-transfected into 293FT packaging cells. The sequence of the miRNA4448 was as follows: gcaattattctttattccaatattataataatcctcgctctataatcataacctaggaaaaaccgggccatacagaggcaggagctgaggggacatagtgaggagtgaccaaaagacaagagtgcgagccttctattatgcccagacagggccaccagagggctccttggtctaggggtaatgccagcgtctgggaagatgcccgttgccaagcagactgtggtctagcggtagcatgtcaaggaaaaacacctgctacttagtagtccctgggggagtttagaga.

The virus-containing supernatant was harvested and filtered 72 h post-transduction. The virus was then used to infect the HCEC line with Polybrene^®^ (Sigma-Aldrich, St. Louis, MO, USA) at a final concentration of 5 µg/ml. Cell culture medium was replaced with fresh complete medium after 24 h of transfection, and the HCEC line was incubated for an additional 24–72 h prior to observing the expression of green fluorescent protein (GFP). Following flow-cytometer (Epics Altra; Beckman Coulter, Inc., Brea, CA, USA) selection for GFP (+) cells, the stable overexpression of miRNA4448 clones was obtained. The analysis software used was Expo32 V1.2 Analysis Multicycle for Windows (Beckman Coulter, Brea, CA, USA).

### Western Blotting

Western blotting was performed using standard protocols. In brief, nuclear proteins were isolated using a nuclear extraction kit (Active Motif, Carlsbad, CA, USA). In addition, cell lysates were separated by sodium dodecyl sulfate polyacrylamide gel electrophoresis, transferred to a polyvinylidene fluoride membrane, and blocked in 5% skimmed milk. Membranes were incubated overnight at 4°C with primary antibodies: anti-rabbit P-P65 (1:1,000; Santa Cruz Biotechnology, Inc., Dallas, TX, USA) or anti-rabbit Lam B1 (1:1,000; Miao Tong Biological Science & Technology Co., Ltd., Shanghai, China). An anti-rabbit secondary antibody (1:2,000; Miao Tong Biological Science & Technology Co., Ltd.) was added to membranes at room temperature (RT) for 1 h. The specific protein bands were visualized using enhanced chemiluminescence plus Western blotting detection reagents (GE Healthcare Life Sciences, Pittsburgh, PA, USA) and an LAS4000 luminescent image analyzer (Kodak, Rochester, NY, USA).

### Immunofluorescent Staining

Human corneal epithelial cells (HCEs) were plated in 24-well plates 24 h before the experiment. Cells were fixed in 4% paraformaldehyde for 30 min at RT and then washed twice with cold PBS. In the membrane rupture period, a cold 1% BSA/PBS solution containing 0.2% Triton X-100 was added to cells for 5 min at 4°C. After three rinses with PBS for 3 min each time, the cells were blocked with 1% BSA/PBS for 1.5 h at RT and then incubated with a P-P65 antibody (1:100; Santa Cruz Biotechnology, Inc.) for 2 h. The cells were washed three times with PBS and then incubated with a FITC-conjugated secondary antibody (1:1,000; Sigma) for 1 h. After rinsing with PBS, the cells were counterstained with DAPI (10 ng/ml) and then mounted. Each sample was analyzed using a fluorescence microscope.

### Luciferase Reporter Assay

To investigate the influence of NFκB on MICA, the binding site between NFκB and MICA was first predicted. The MICA promoter sequence contains a putative binding site (178–192: atcggaatcacctag) with NFκB. The wild type (WT) or mutant MICA promoter were cloned into the pGL4.10 plasmid (Obio, Shanghai, China). The WT construct contained the potential binding sequence, and the mutant construct contained the sequence with a –ggaa– and –cc– deletion. The NFκB 3Flag sequence was constructed and cloned into the pcDNA3.1 vector (Obio, Shanghai, China). 293 T cells grown in 96-well plates were harvested for assays 48 h after transfection with pcDNA3.1-NFKB-3Flag and either the pGL4.10-MICA promoter-WT or pGL4.10-MICA promoter-mutant *via* Lipofectamine 2000 (Invitrogen). The firefly and Renilla luciferase activities were measured using a dual-luciferase reporter assay system (Promega Corporation) with a microplate luminometer, and each sample’s luciferase activity was normalized to that of Renilla.

### MICA Plasmid Transfection

We used two types of MICA plasmids in our research. In order to overexpress MICA in the HCECs, in Annexin V-PI staining, we used pcDNA3.1-MICA and pcDNA3.1-empty plasmid (GeneCopoeia, Rockville, MD, USA), which were transfected into HCECs using Lipofectamine 3000 (Invitrogen). In the Caspase and Survivin mRNA expression study, a MICA ORF clone was transfected into the pcDNA3.1-GFP plasmid, and the pcDNA3.1-GFP-MICA or empty control vector was transfected into HCECs using Lipofectamine 2000 (Invitrogen). The cells were cultured for 24–72 h following transfection, and flow-cytometry was used to obtain GFP (+) cells.

### Annexin V/PI Staining

After washing three times with PBS, HCECs were treated with 0.25% trypsin to prepare the cell suspension. FBS was used to neutralize the trypsin, and the cells were then rinsed three additional times followed by centrifugation for 5 min at 1,000 rpm. Next, 300 µl of binding buffer was added to suspend the cells, and the cells were incubated with Annexin V/PI (5 µl, 20 min for Annexin V and 5 µl, 5 min for PI). The apoptosis rate of each group was analyzed using flow cytometry.

### Statistical Analysis

Each experiment was performed in triplicate, and the data were expressed as mean ± SD. Differential miRNA expression, miRNA4448 expression after pLenti-CMV-GFP-miR4448 transfection, and the CCK8 proliferation assay were analyzed using a Student’s *t* test (SPSS 13.0, USA). Differences among more than two groups, such as the MICA and P65 expression, luciferase reporter assay and Caspase and Survivin mRNA expression, were evaluated using a one-way ANOVA. *P* < 0.05 was considered significant.

## Results

### IFN-γ Upregulated MICA Expression in HCECs

**Figure 1:**
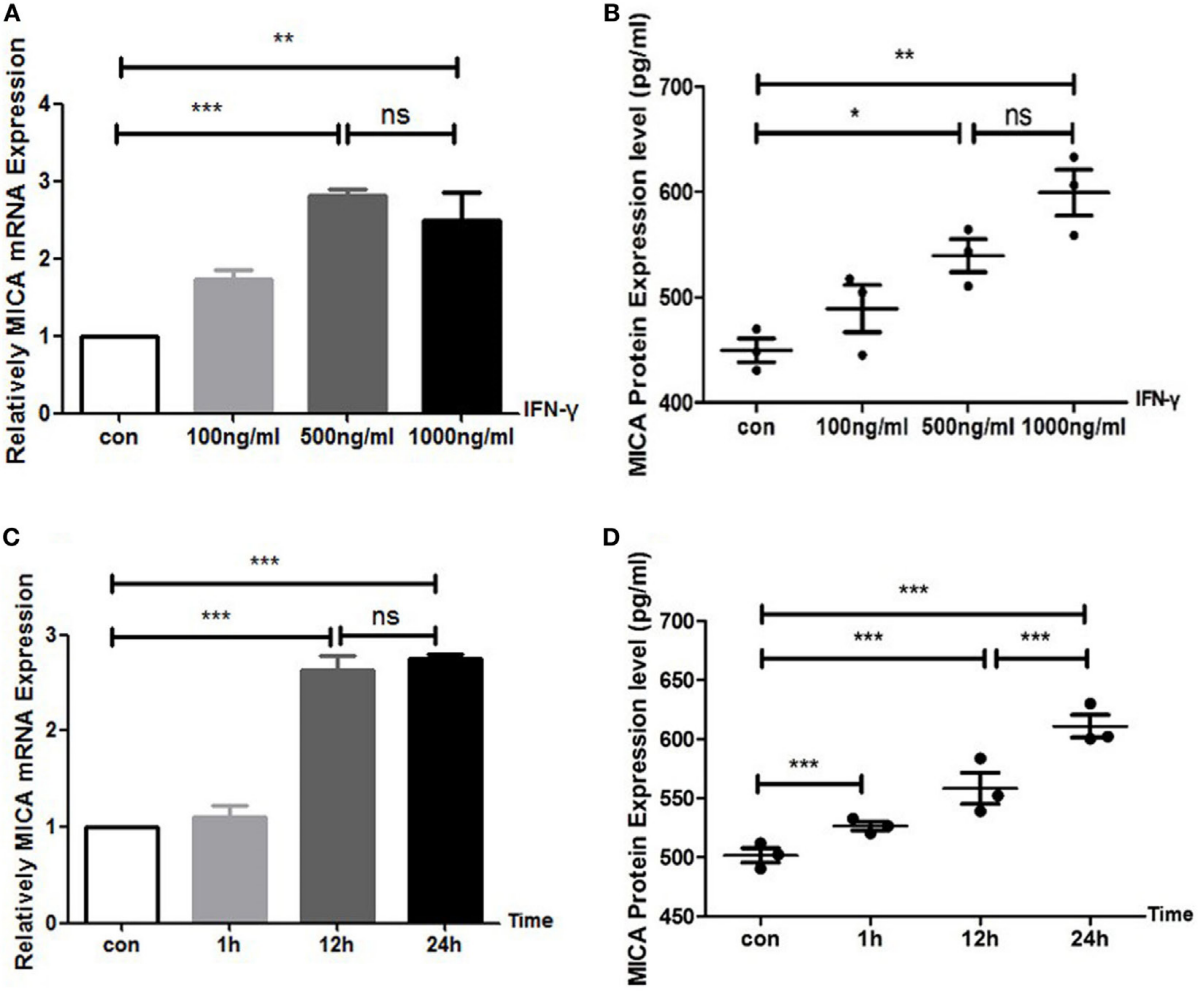
IFN-γ increases MICA expression levels in human corneal epithelial cells. **(A)** RT-PCR analysis of MICA mRNA expression and **(B)** enzyme-linked immunosorbent assay analysis of the MICA protein expression levels after IFN-γ treatment for 24 h. **(C)** The expression of MICA mRNA and **(D)** protein in cells treated with 500 ng/ml IFN-γ. Data are shown as the mean ± SD. *p < 0.05; **p < 0.01; ***p < 0.001 vs. untreated control. Con, control; ns, not significant.

With the IFN-γ treatment for 24 h, the expression levels of MICA protein and MICA mRNA in HCECs were upregulated in a concentration-dependent manner (Figure 1). MICA mRNA and protein expression levels reached their peak at 12 and 24 h after IFN-γ (500 ng/ml) treatment, respectively (Figure 1). Therefore, treating HCECs with 500 ng/ml of IFN-γ for 24 h was identified as the optimal condition. Additionally, the CCK8 assay showed that 500 ng/ml of IFN-γ treating HCECs for 24 h had no toxic effect on cells (450 nm optical density value: con VS IFN-γ: 1.10 ± 0.10 vs 1.12 ± 0.07, *p* > 0.05).

### Differential miRNA Expression in HCECs Following IFN-γ Treatment

**Figure 2:**
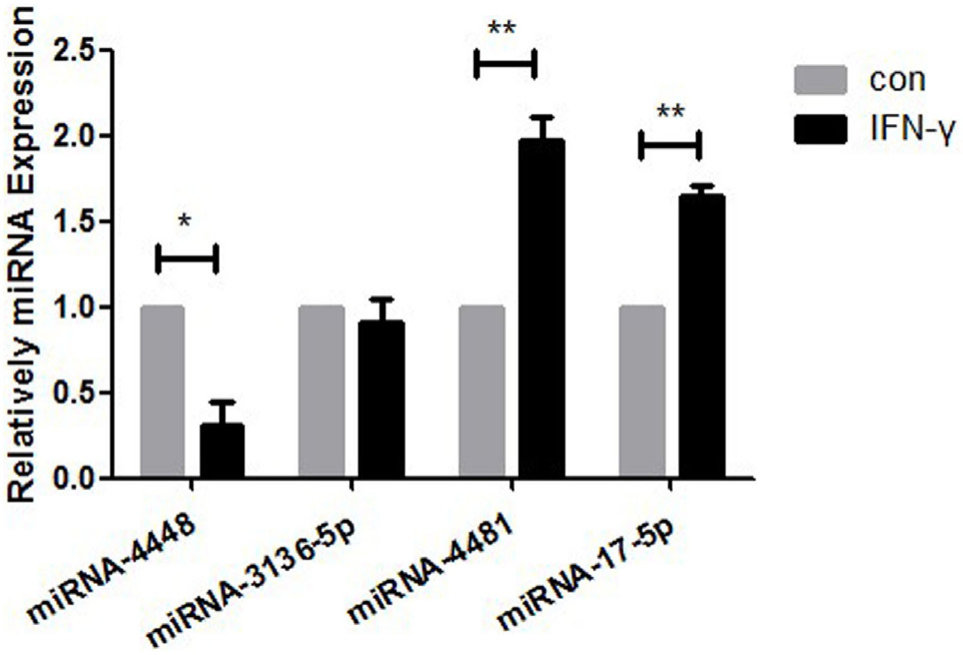
RT-PCR measurement of the expression levels of hsa-miR4448, hsa-miR3136-5p, hsa-miR4481, and hsa-miR17-5p in human corneal epithelial cells following 24-h treatment with 500 ng/ml IFN-γ. Data are shown as the mean ± SD. *p < 0.05; **p < 0.01 vs. untreated control. Con, control; miRNA, microRNA.

There were two upregulated miRNAs and four downregulated miRNAs in the IFN-γ-treated HCECs according to the Affymetrix Gene Chip miRNA array. GO and IPA analysis showed that four of the six miRNAs—hsa-miR-17-5p, hsa-miR-4448, hsa-miR-4481, and hsa-miR-3136-5p—might regulate several critical pathways that influence MICA expression. The regulator including the NFκB, human protection of telomeres 1, cluster of differentiation 28 (CD28), and mitogen-activated protein kinase (MAPK) pathways. According to qRT-PCR (Figure 2; Table 2), the fold change values for the miRNA in the IFN-γ group were as follows: miRNA4448, 0.31 ± 0.14-fold (*p* < 0.05); miRNA3136-5p, 0.91 ± 0.13-fold (*p* > 0.05); miRNA4481, 1.98 ± 0.33-fold (*p* < 0.05); and miRNA17-5p, 1.64 ± 0.19-fold (*p* < 0.05). Among them, miRNA4448, which displayed the most significant change, was selected for further investigation.

**Table 2:** Differential miRNA expression in human corneal epithelial cells following IFN-γ treatment.

Variation trend (IFN-γ/con)	miRNA
Increase	hsa-miR-17-5p, hsa-miR-4481
Decrease	hsa-miR-3136-5p, hsa-let-7a-2-3p, hsa-miR-6823-5p, hsa-miR-4448

### Overexpression of miRNA4448 by Transfection of pLenti-CMV-GFP-miR-4448 in HCECs

**Figure 3:**
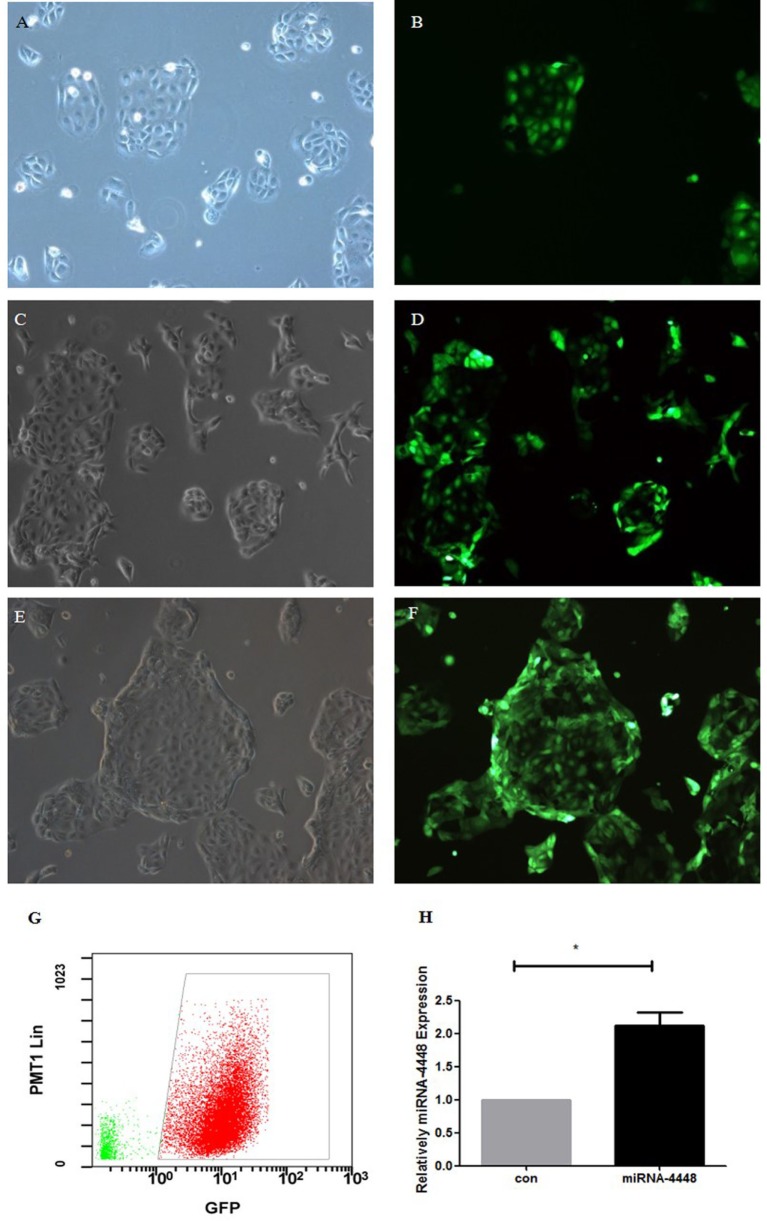
Overexpression of miRNA4448 in pLenti-CMV-GFP-miR-4448 human corneal epithelial cells (HCECs). The cell morphology and GFP fluorescence was examined after pLenti-CMV-GFP-miR-4448 was transfected for 24 h **(A,B)**, 48 h **(C,D)**, and 72 h **(E,F)** and imaged using phase contrast microscopy (left column) and fluorescent microscopy (left column). **(G)** Flow cytometry was used to sort the GFP (+) cells to establish the pLenti-CMV-GFP-miR-4448 and the pLenti-CMV-GFP-NC HCEC lines, which contained GFP (+) cells at concentrations up to 96.3%. **(H)** RT-PCR demonstrated the expression levels of miRNA4448 were upregulated in the pLenti-CMV-GFP-miR-4448 HCEC line. *p < 0.05 vs. pLenti-CMV-GFP-NC. GFP, green fluorescent protein.

Human corneal epithelial cells revealed normal cell morphology up to 72 h after transfection with pLenti-CMV-GFP-miR-4448 or pLenti-CMV-GFP-NC. GFP fluorescence showed a small, medium, and large amount of expression at 24, 48, and 72 h post-transfection, respectively, and the fluorescence intensity increased in a time-dependent manner (Figure 3). Furthermore, qRT-PCR indicated that miRNA4448 expression was 2.118 ± 0.29 times increased in pLenti-CMV-GFP-miR-4448 HCEC lines (*p* < 0.05; Figure 3).

### MiRNA4448 Decreased MICA mRNA and Protein Expression Levels in HCEC

**Figure 4:**
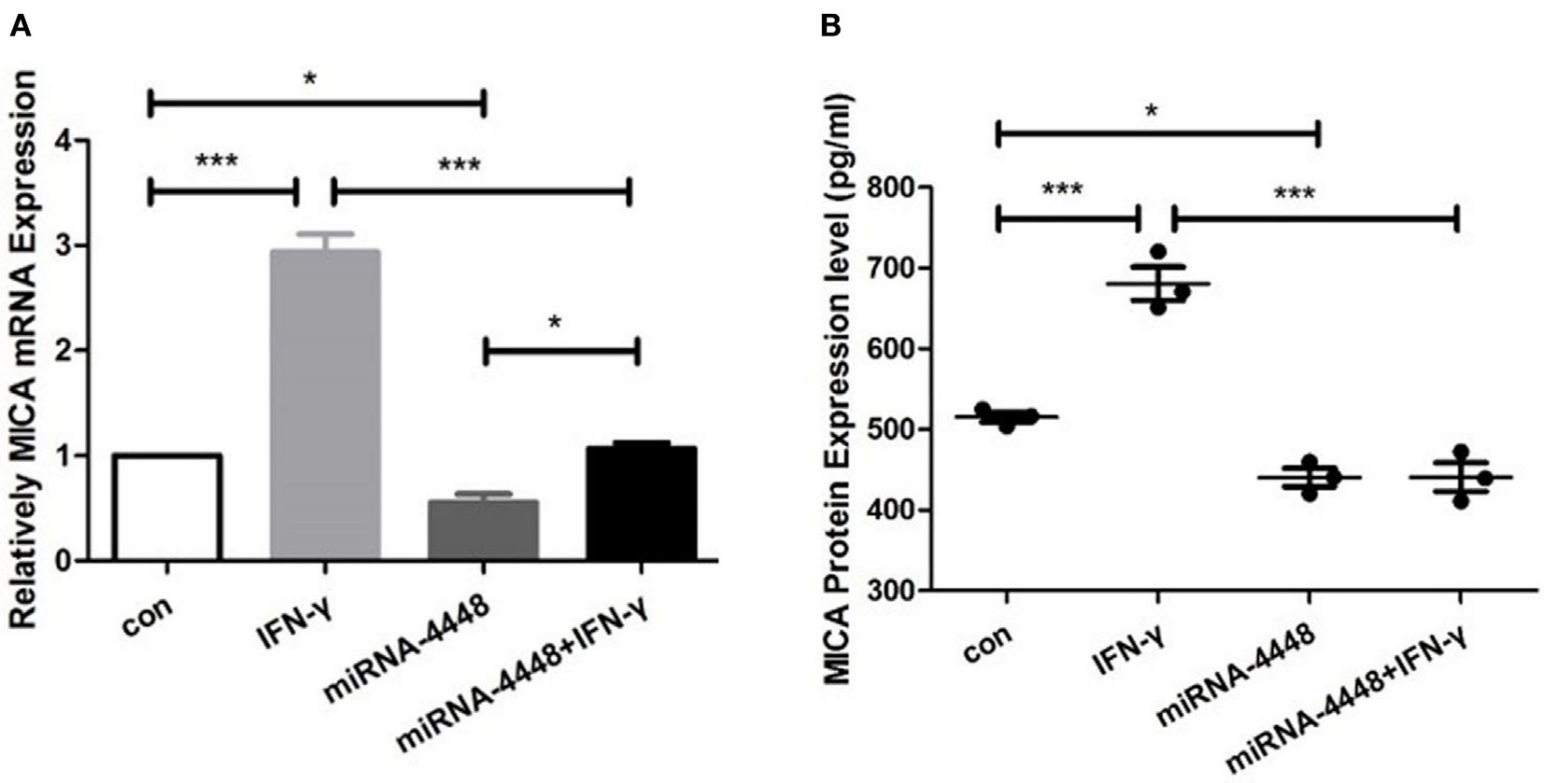
miRNA4448 is IFN-γ responsive, and the overexpression of miRNA4448 decreased MICA mRNA and protein expression levels in human corneal epithelial cells (HCECs). Based on **(A)** qRT-PCR **(B)** and enzyme-linked immunosorbent assay analysis, IFN-γ increased MICA mRNA and protein expression levels in the pLenti-CMV-GFP-NC HCEC line. Transfecting pLenti-CMV-GFP-miR-4448 decreased MICA levels compared with transfecting pLenti-CMV-GFP-NC, whereas IFN-γ treatment partially reversed the MICA mRNA expression. *p < 0.05; ***p < 0.001. Con and IFN-γ: pLenti-CMV-GFP-NC HCEC line. miRNA 4448 and miRNA 4448 + IFN-γ: pLenti-CMV-GFP-miR-4448 HCEC line.

In the pLenti-CMV-GFP-NC HCEC line, MICA mRNA levels increased 2.94 ± 0.24 times, and protein levels increased 1.32 ± 0.08 times after IFN-γ treatment (Figure 4). Overexpression of miRNA4448 significantly decreased the MICA mRNA and protein expression to 55.53 and 85.48%, respectively, compared to the pLenti-CMV-GFP-NC HCEC lines (Figure 4). Furthermore, IFN-γ treatment could partially reverse the MICA mRNA expression (Figure 4).

### IFN-γ and miRNA4448 Mediated NFκB/P65 Expression in HCECs

**Figure 5:**
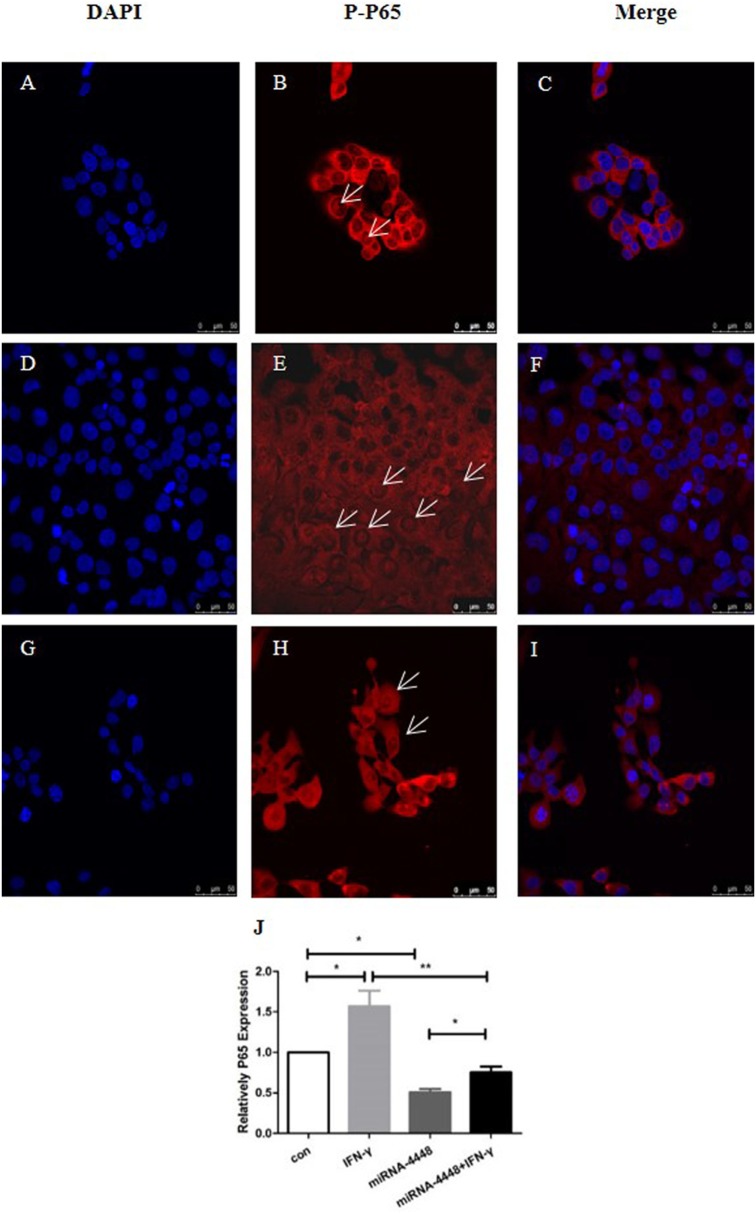
IFN-γ and miRNA4448 mediated NFκB/P65 expression in human corneal epithelial cells (HCECs). DAPI (blue) and the activation of P65 (red) was determined by immunofluorescent staining. **(A–C)** There were a few P-P65 present in the nucleus of the control group **(D–F)**, while IFN-γ treatment increased the number of P-P65-positive nuclei. **(G–I)** The overexpression of miRNA4448, however, decreased the P-P65-positive nuclei, indicating the inhibition of P65. **(J)** P65 mRNA expression levels in the pLenti-CMV-GFP-NC and pLenti-CMV-GFP-miR-4448 HCEC lines was measured using RT-PCR. *p < 0.05; **p < 0.01; Con and IFN-γ: pLenti-CMV-GFP-NC HCEC line. miRNA 4448 and miRNA 4448 + IFN-γ: pLenti-CMV-GFP-miR-4448 HCEC line.

Based on immunofluorescent staining, IFN-γ treatment markedly promoted the phosphorylation and nuclear translocation of P65, while this effect was substantially inhibited by overexpression of miRNA4448 (Figure 5; Table 3). We also examined the expression of P65 using qRT-PCR. IFN-γ treatment upregulated P65 mRNA expression levels by 1.57 ± 0.27 fold, while the overexpression of miRNA4448 downregulated P65 mRNA levels by 50.9% compared to the control group (Figure 5). P65 mRNA expression levels were partially reversed to 75% in the miRNA4448 overexpression group following IFN-γ treatment (Figure 5). Overall, these results suggest that IFN-γ activated NFκB/P65 through the regulation of miRNA4448 in HCECs.

**Table 3:** NFκB-p65 positive and negative cell numbers.

Cell type (number/frame)	Control	IFN-γ	miRNA4448	*p*-Value
NFκB-p65(+)	4.6 (14.3%)	19 (59.4%)	3 (9.7%)	0.018
NFκB-p65(−)	28 (85.7%)	39 (40.6%)	28.7 (90.3%)	

### MICA mRNA and Protein Expression Levels Decreased After PDTC Treatment

**Figure 6:**
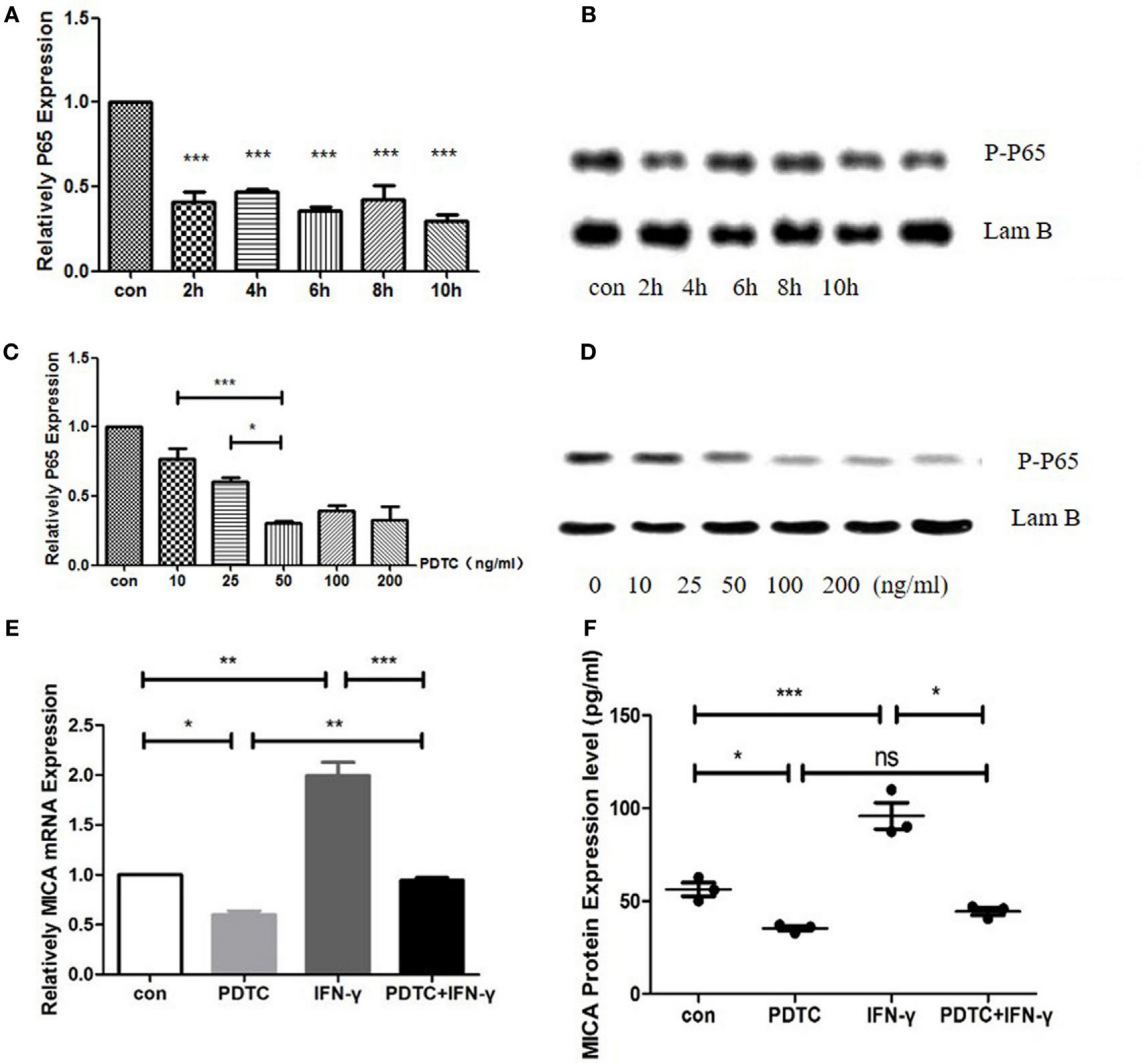
Inhibition of NFκB by pyrrolidine dithiocarbamate (PDTC) decreased MICA expression levels. **(A)** RT-PCR analysis of the MICA mRNA levels **(B)** and enzyme-linked immunosorbent assay (ELISA) analysis of the MICA protein expression levels in cells treated with 100 ng/ml of PDTC. **(C)** The expression of MICA mRNA levels **(D)** and protein levels after PDTC treatment for 2 h. **(E,F)** PDTC decreased both MICA mRNA and protein expression levels in human corneal epithelial cells, as demonstrated by RT-qPCR and ELISA, respectively. IFN-γ treatment significantly upregulated the MICA mRNA expression levels compared to the PDTC group.*p < 0.05; **p < 0.01; ***p < 0.001.

When HCECs was treated with PDTC for 2 h, the expression of P65 mRNA decreased significantly to 41% compared to the control group (*p* < 0.05, Figure 6). As time progressed, there was no significant difference between the 4-, 6-, 8-, and 10-h treatment groups compared to the 2-h treatment group (*p* > 0.05, Figure 6). Meanwhile, the P-P65 protein levels decreased the most in the 2-h treatment group. The qRT-PCR analysis showed that when the PDTC concentration increased from 0 to 50 ng/ml, P65 mRNA expression was gradually inhibited, but there was no statistical difference among the 50, 100, and 200 ng/ml groups (*p* > 0.05, Figure 6). P65 mRNA levels decreased to 30.8% in the 50 ng/ml PDTC group compared to the control group. Moreover, the downregulation of the P-P65 protein was the most significant in the 50 ng/ml group (Figure 6). Next, 500 ng/ml IFN-γ was used to treat cells for another 24 h. This showed that IFN-γ could significantly upregulate the expression of MICA mRNA levels by 1.58 times compared to the PDTC group.

### NFκB Action on the MICA Promoter Region

**Figure 7:**
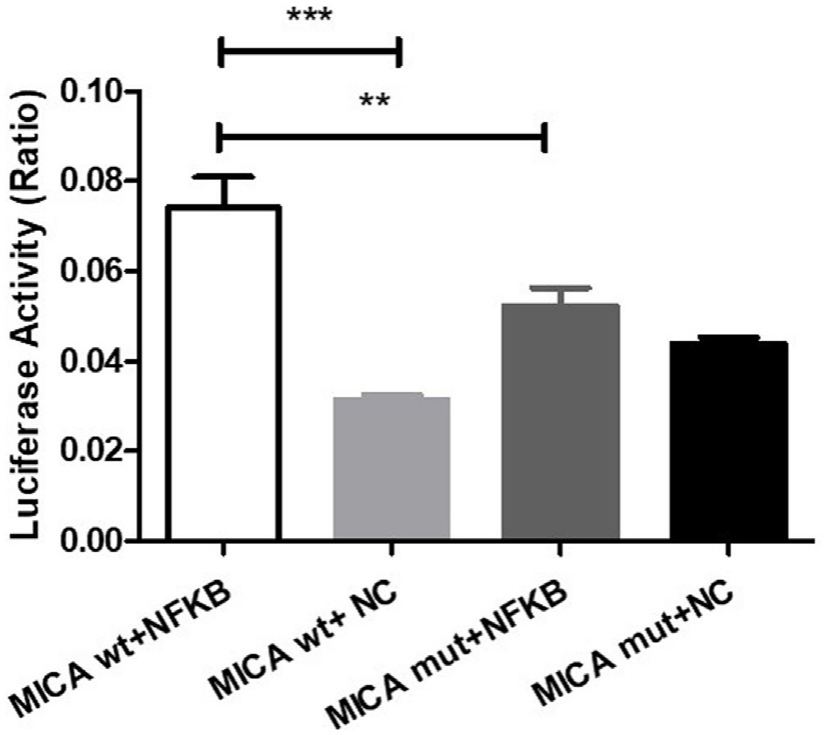
NFκB action on the MICA promoter region. Luciferase reporter assay of 293 T cells transfected with pGL4.10-MICA promoter-WT or pGL4.10-MICA promoter-mutant together with cDNA3.1-NFκB-3Flag or NC. **p < 0.01; ***p < 0.001. WT, wild type; mut, mutant; NC, negative control; ns, not significant.

cDNA3.1-NFκB-3Flag and either pGL4.10-MICA promoter-WT or pGL4.10-MICA promoter-mutant were co-transfected to investigate the potential function of NFκB on MICA. The results show that NFκB significantly increased the luciferase activity of pGL4.10-MICA promoter-WT in the 293 T cells (*p* < 0.001; Figure 7), whereas it had no significant effect on the mutant group (*p* > 0.05; Figure 7). This indicates that NFκB can act on the MICA promoter region and promote MICA gene expression.

### Overexpression of MICA by Transfection of pcDNA3.1-GFP-MICA in HCECs

pcDNA3.1-GFP-MICA or pcDNA3.1-GFP-NC was transfected into HCECs for 24, 48, or 72 h. The expression of MICA mRNA was 1.836 ± 0.155 times greater in the pcDNA3.1-GFP-MICA line than in the NC group, as determined by qRT-PCR (*p* < 0.05). According to the ELISA results, the MICA protein levels were significantly upregulated by 1.54 times compared to the NC group. Overall, the transfection of pcDNA3.1-GFP-MICA in the HCEC line resulted in increased MICA mRNA and protein levels.

### MICA-Enhanced HCEC Apoptosis Mediated by NK Cells and CD8 + T Cells

**Figure 8:**
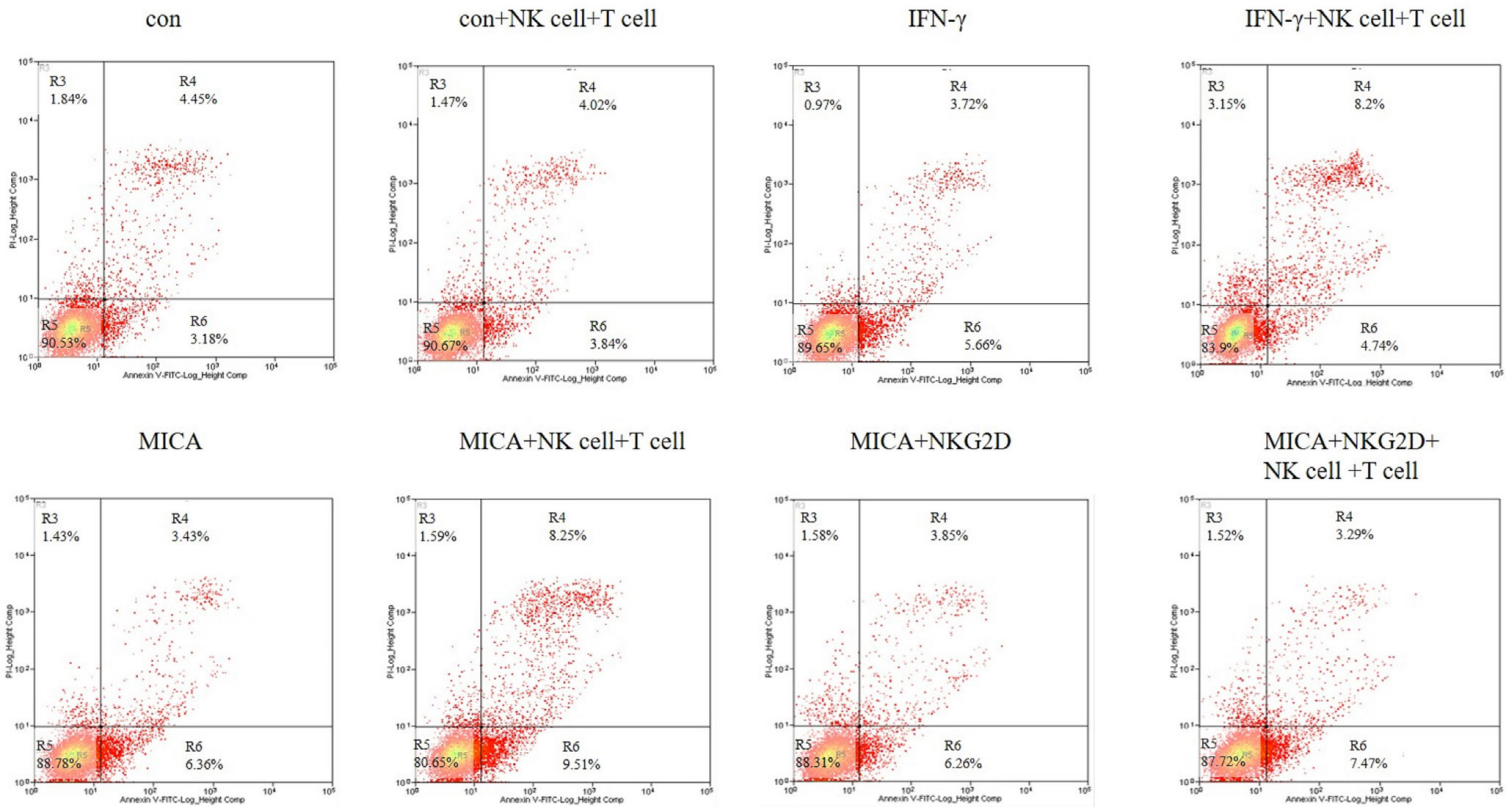
Effect of IFN-γ treatment and MICA plasmid transfected on human corneal epithelial cells apoptosis in terms of Annexin-V/PI staining. Numbers represent the percentage of cells in each quadrant.

To confirm the apoptosis effect of MICA, Annexin V/PI staining was used to quantify the number of apoptotic cells after HCECs were cocultured with allergic NK cells and CD8+ T cells. As shown in Figure 8, the ratio of late apoptotic cells increased from 4.45 to 8.20 and 8.25% when HCECs were incubated with IFN-γ or transfected with MICA-plasmid compared with the control group. On the contrary, when HCECs were pre-incubated with NKG2D-mAb, the percentage of late apoptotic cells decreased significantly to 3.29%. Collectively, these results demonstrate that the interaction between HCECs and NK (CD8+ T) cells upon MICA stimulation results in the late apoptosis of HCEC cells and is mediated by MICA–NKG2D interaction.

### Survivin Expression Levels Decreased and Caspase3 Expression Levels Increased After MICA Overexpression

**Figure 9:**
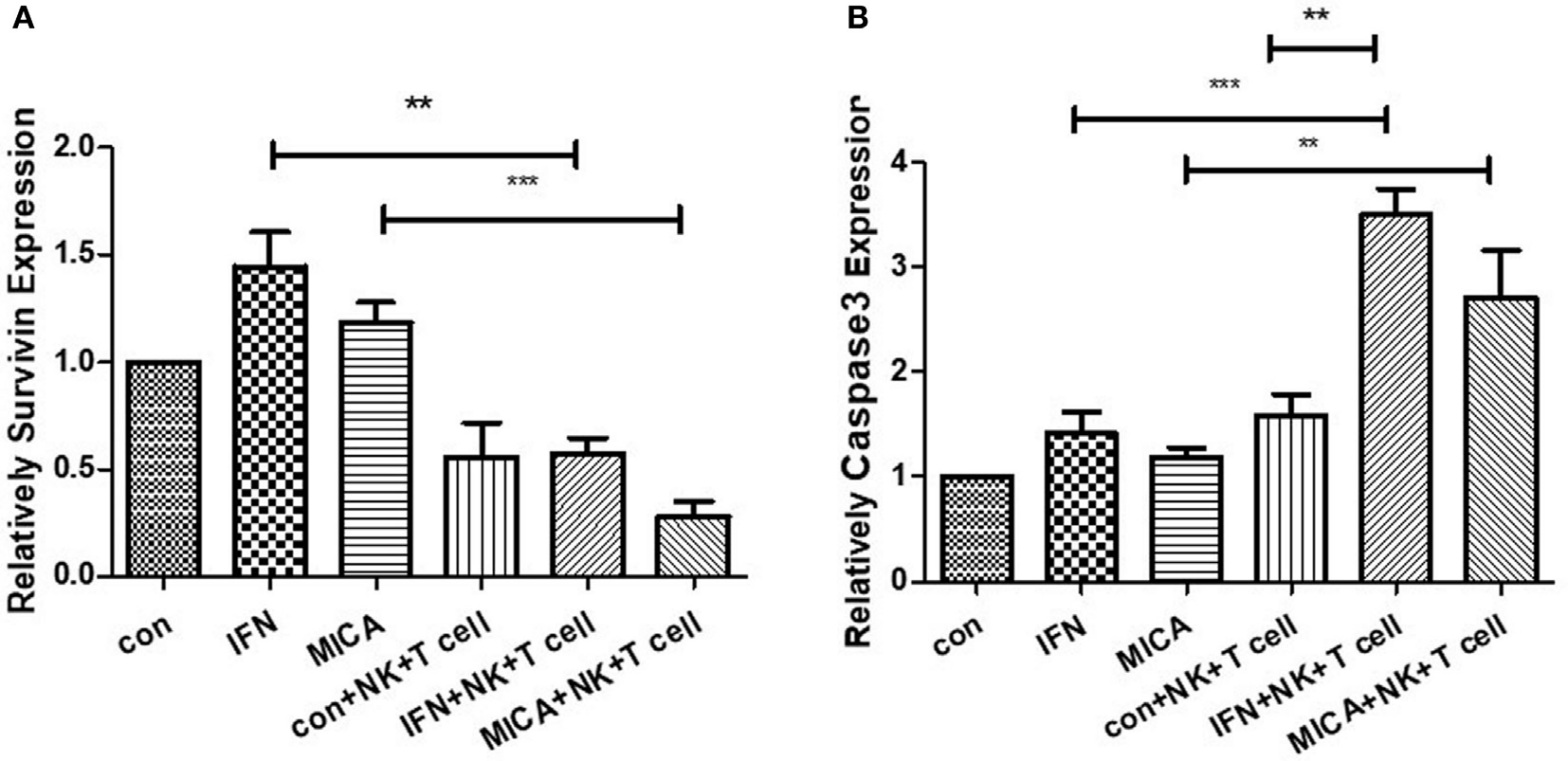
Survivin expression **(A)** decreased and Caspase3 expression **(B)** increased with MICA overexpression. RT-qPCR measured the Survivin and Caspase 3 mRNA expression levels after pcDNA3.1-GFP-MICA transfection or IFN-γ treatment. **p < 0.01; ***p < 0.001.

As determined by RT-qPCR analysis, in pcDNA3.1-GFP-MICA-transfected or IFN-γ treatment groups, the expression level of Survivin was obviously decreased and that of Caspase3 was significantly increased when HCECs were cocultured with NK cells and CD8+ T cells (*p* < 0.01; Figure 9). The overexpression of MICA increased NK cells and CD8+ T cell-induced HCEC cell apoptosis *via* the regulation of Survivin and Caspase3.

## Discussion

MICA, a non-classical MHC molecule, is expressed in corneal epithelial and endothelial cells. To date, little is known about the expression of MICA and its regulation mechanisms in relation to corneal immunity. In this study, it was shown that IFN-γ can regulate miRNA4448 and NFκB, thereby increasing MICA expression, which in turn enhanced NK and CD8+ T cell-mediated cytotoxicity against the corneal epithelium.

miRNA4448 is located on chromosome 3q27.1, and its expression has been verified in tumor cells and lymphocytes. The upregulation of miRNA4448 expression in the MCF-7 cell line is related to drug resistance in tumor cells (13), and in glioblastoma multiforme miRNA4448 is associated with the survival period (15). This study was the first to report miRNA4448 expression in HCECs. Previous studies demonstrated that IFN-γ mainly played a role in activating signaling pathways, such as the janus kinase (JAK), signal transducer and activator of transcription, and Akt signaling pathways (16–18). We found that IFN-γ upregulated MICA expression levels through the inhibition of miRNA4448. Overexpression of miRNA4448 inhibited MICA levels, whereas IFN-γ stimulation partially restored MICA expression, demonstrating that miRNA4448 acted downstream of IFN-γ and was involved in MICA regulation in HCECs.

We further found that the regulation of MICA by IFN-γ was concentration-dependent. When the dose of IFN-γ was less than 500 ng/ml, the MICA expression levels increased significantly. If the IFN-γ concentration was greater than 500 ng/ml; however, the MICA expression levels did not change, indicating that a small-to-medium dose of IFN-γ significantly elevated MICA expression levels. After IFN-γ treatment, MICA protein levels were upregulated up to 24 h posttreatment, which indicated that 500 ng/ml and 24 h of treatment were the optimal conditions for IFN-γ in HCECs.

To date, little is known about the transcriptional regulation of the MICA gene. DNA methylation and transcription factors, such as specific protease 1 and specific protease 3, play an important role in MICA transcriptional regulation (19). We proved that miRNA4448 affects MICA expression levels through the regulation of NFκB/P65. NFκB is widely involved in multiple pathophysiological processes, such as corneal development (20), wound healing (21), and neovascularization (22–24). We found that the overexpression of miRNA4448 could downregulate NFκB and prevent its transcription into the nucleus. IFN-γ can inhibit miRNA4448 and partly restore NFκB expression, indicating that miRNA4448 acts as a downstream factor in the IFN-γ signaling pathway and negatively regulates NFκB expression. PDTC, a potential inhibitor of NFκB, can inhibit the role of NFκB by inhibiting IκB phosphorylation, thus blocking NFκB translocation to the nucleus and reducing the expression of downstream cytokines (25, 26). In this study, we found PDTC could partially inhibit the upregulation of the MICA expression levels induced by IFN-γ, indicating that IFN-γ regulated the expression of MICA through NFκB.

It has been found that TNF-α and γ-rays can increase MICA expression by activating the NFκB and JNK pathways in acute graft-versus-host disease, leading to graft damage (27). DNA damage (28) and the MAPK pathway (29) can regulate MICA gene expression by activating NFκB, indicating that NFκB play a critical role in multiple pathways regulating MICA. NFκB has been related to acute ischemic injuries and the ischemic inflammatory process (30), showing how this protein might affect graft survival. However, whether NFκB affects corneal graft function has yet to be reported.

Pyrrolidine dithiocarbamate partially inhibited the upregulation of MICA, which can be explained in several ways. First, the commonly used methods for inhibiting NFκB activation involve inhibiting IκB kinase activity, increasing IκB synthesis, and inhibiting IκB degradation. The mechanism of action of PDTC is the inhibition of IκB degradation, thereby preventing NFκB activation. In addition to promoting the release of NFκB by degrading IκB, IFN-γ might directly activate NFκB p65 phosphorylation without relying on IκB (31). Second, gene expression is regulated by a complex signaling pathway, and the same stimulation might activate different signaling pathways in various cell types. Therefore, in corneal epithelial cells, there might be other signaling pathways and transcription factors that upregulate MICA upon induction via IFN-γ treatment.

IFN-γ is involved in the regulation of NK cells and cytotoxic lymphocytes in antiviral immunity (32, 33). Moreover, IFN-γ promotes cytotoxic lymphocyte lysis *via* MICA regulation in tumor cells (34). Previous studies have shown a higher susceptibility to cytotoxicity mediated by NK cells in corneal epithelium transfected with C1R-MICA plasmids (35). Consistent with previous studies, we discovered that NK cells and CD8+ T cells can cause significant apoptosis in MICA-transfected or IFN-γ-treated HCECs, indicating that IFN-γ may promote corneal immunity through MICA regulation. IFN-γ and NK cells can directly kill target cells in natural killer group 2 member D (NKG2D)-, tumor necrosis factor-related apoptosis-inducing ligand (TRAIL)-, and granzyme-dependent manners (36, 37). In our experiment, we found the late apoptosis of HCECs was inhibited when we pre-treated cells with NKG2D-mAbs. Therefore, we think that IFN-γ upregulated MICA in HCECs and that MICA stimulates the killing effect of NK and CD8^+^ T cells *via* MICA–NKG2D interaction. In addition, we provided evidence for the first time that Survivin downregulation and Caspase3 upregulation after MICA overexpression could be a possible mechanism for MICA involvement in HCEC apoptosis.

We note that there are some limitations to this study. First, the study was conducted *in vitro*, and further investigation should be done to verify the function and mechanism of IFN-γ on MICA expression *in vivo*. Second, the regulatory mechanism of miRNA4448 on NFκB requires further study in HCECs.

Overall, we discovered that IFN-γ can inhibit miRNA4448 and regulate NFκB, thereby increasing MICA expression levels and in turn enhancing CD8+ T cell and NK cell-mediated cytotoxicity against corneal epithelium via MICA–NKG2D interaction. Attention should be paid to MICA expression levels to avoid potential damage to HCECs in corneal transplantations, corneal keratitis, and other corneal disorders. Moreover, the regulation of HCECs upon IFN-γ and MICA treatment leads to new insights about corneal immune status.

## Author Contributions

DW, JX, and JH designed the experiments. DW, JZ, TQ, and YD performed the experiments and the data analyses. DW and AM wrote the manuscript. JX and JH helped perform the analysis with constructive discussions.

## Conflict of Interest Statement

No conflict of interest exists in the submission of this manuscript. Furthermore, the manuscript is approved by all authors for publication.
